# Generation of corrected hiPSC clones from a Cornelia de Lange Syndrome (CdLS) patient through CRISPR-Cas-based technology

**DOI:** 10.1186/s13287-022-03135-0

**Published:** 2022-09-02

**Authors:** Alessandro Umbach, Giulia Maule, Eyemen Kheir, Alessandro Cutarelli, Marika Foglia, Luca Guarrera, Luca L. Fava, Luciano Conti, Enrico Garattini, Mineko Terao, Anna Cereseto

**Affiliations:** 1 Department CIBIO, University of Trento, Via Sommarive 9, 38123 Povo, Italy; 2 Laboratory of Molecular Biology, Istituto Di Ricerche Farmacologiche Mario Negri IRCCS, Via Mario Negri 2, 20156 Milan, Italy

**Keywords:** Cornelia de Lange Syndrome (CdLS), CRISPR-Cas9, Homology Directed Repair (HDR), Base editor, Prime editor, Patient-derived hiPSCs, Nipped-B-like protein (NIPBL), Isogenic cell line

## Abstract

**Background:**

Cornelia de Lange syndrome (CdLS) is a rare multisystem genetic disorder which is caused by genetic defects involving the Nipped-B-like protein (*NIPBL*) gene in the majority of clinical cases (60–70%). Currently, there are no specific cures available for CdLS and clinical management is needed for life. Disease models are highly needed to find a cure. Among therapeutic possibilities are genome editing strategies based on CRISPR-Cas technology.

**Methods:**

A comparative analysis was performed to test the most recent CRISPR-Cas technologies comprising base- and prime-editors which introduce modifications without DNA cleavages and compared with sequence substitution approaches through homology directed repair (HDR) induced by Cas9 nuclease activity. The HDR method that was found more efficient was applied to repair a CdLS-causing mutation in the *NIPBL* gene. Human-induced pluripotent stem cells (hiPSCs) derived from a CdLS patient carrying the c.5483G > A mutation in the NIPBL were modified through HDR to generate isogenic corrected clones.

**Results:**

This study reports an efficient method to repair the *NIPBL* gene through HDR mediated by CRISPR-Cas and induced with a compound (NU7441) inhibiting non-homologous end joining (NHEJ) repair. This sequence repair method allowed the generation of isogenic wild-type hiPSCs clones with regular karyotype and preserved pluripotency.

**Conclusions:**

CdLS cellular models were generated which will facilitate the investigation of the disease molecular determinants and the identification of therapeutic targets. In particular, the hiPSC-based cellular models offer the paramount advantage to study the tissue differentiation stages which are altered in the CdLS clinical development. Importantly, the hiPSCs that were generated are isogenic thus providing the most controlled experimental set up between wild-type and mutated conditions.

**Supplementary Information:**

The online version contains supplementary material available at 10.1186/s13287-022-03135-0.

## Background

Cornelia de Lange syndrome (CdLS) is a rare multisystem genetic disorder affecting 1/10,000–1/30,000 live births. It has a wide range of clinical manifestations, including pre- and postnatal development retardation, psychomotor delay, feeding difficulties, behavioral problems, facial dysmorphism, upper limb malformations, congenital heart defects, gastrointestinal dysfunction, hearing loss and ophthalmologic manifestations [1, 2]. Currently, there are no specific cures available for CdLS, and clinical management is needed for life.

CdLS is linked to mutations in genes coding for subunits or regulators of the cohesin complex which is involved in sister chromatid cohesion, chromosome condensation, DNA repair and transcriptional regulation [3–5]. Approximately, 60–70% of CdLS diagnoses are associated with mutations in the *NIPBL* gene [6–9]. *NIPBL* encodes for a protein called Delangin, which is the human homologue of the yeast and fly sister chromatid cohesion protein 2 (SCC2), and together with SCC4 (MAU2 in mammals) forms the cohesin loading complex (or kollerin), required for cohesin loading onto DNA [10]. Increasing evidence suggests that Delangin deficits are linked to chromatin architecture alterations and transcriptional deregulation [11]. To study the function of the *NIPBL* gene and its role in CdLS development, various cellular and animal models have been developed. These include human B lymphoblastoid cell lines (LCL), human-induced pluripotent stem cells (hiPSCs), *Drosophila melanogaster*, *Danio rerio* and mice [12–15]. In particular, patient-derived hiPSCs are optimal tools to study *NIPBL* genetic defects toward the development of therapeutic strategies by means of drug screening and drug repurposing [16]. Yet, no isogenic hiPSCs have been produced so far allowing to perform better control experiments.

The advancement of technologies for genome manipulations, in particular CRISPR-Cas systems, highly facilitated the generation of more refined disease models and is presently opening-up new perspectives for genetic treatments of diseases. Following the technological progression diverse CRISPR-Cas strategies have been developed which can be grouped in two main classes: (1) modifications introduced through Cas induced DNA double strand break repair (DSB) or (2) modifications induced by functional modules (deaminase or reverse transcriptase) fused to a Cas9 nickase, which promotes single strand cleavages [17–20].

In this study, we tested both approaches and demonstrated that CRISPR-Cas mediated homology directed repair (HDR) obtained with Cas9 nucleases efficiently and precisely corrects the c.5483G > A *NIPBL* point mutation in hiPSCs derived from a CdLS patient. This editing strategy allowed to generate isogenic hiPSCs, wild-type and mutated cell lines, which will be instrumental to study the molecular mechanisms leading to CdLS and to develop new therapeutic approaches.

## Methods

### Plasmids and oligonucleotides

pcDNA3—*NIPBL* WT was obtained by amplifying a portion of the coding sequence of *NIPBL* containing exons 27, 28, 29 and 30 with primers For BamHI and Rev EcoRI (all primers are listed in Additional file 2: Table S3) and cloning into a previously published pcDNA3 plasmid [21]. The *NIPBL* c.5483 G > A mutation was generated by amplifying two amplicons from pcDNA3 – *NIPBL* WT with For BamHI and Rev BsmBI—mut, For BsmBI—mut and Rev EcoRI, respectively. The two amplicons were digested with BsmBI, ligated and amplified using For BamHI and Rev ECORI primers to generate an amplicon containing the *NIPBL* c.5483G > A mutation, pcDNA3—*NIPBL* c.5483G > A was generated by cloning the amplicon containing the *NIPBL* c.5483G > A mutation into the pcDNA3 plasmid, using BamHI and ECORI as restriction sites.

SpCas9 was expressed from the pX-SpCas9 plasmid, which was obtained by removal of an NdeI fragment including the sgRNA expression cassette from pX330 (a gift from Feng Zhang, Addgene # 42230). SpCas9-NG and SpCas9-VQR were obtained by site directed mutagenesis of pX-SpCas9 plasmid.

Plasmid pY108 (lenti-EnCpf1) was obtained by cloning enAsCas12a [22] into the pY108 (lenti-AsCpf1) (Addgene plasmid # 84739; http://n2t.net/addgene:84739; RRID:Addgene_84739).

ABEmax-SaCas9 plasmid was obtained by subcloning the SaCas9 nickase sequence into the pCMV-ABEmax (Addgene plasmid # 112095; http://n2t.net/addgene:112095; RRID:Addgene_112095). ABE8e-SaCas9 and ABE8.20m-SaCas9 were obtained by cloning the sequences of the adenine deaminases (GeneScript) into the ABEmax-SaCas9 plasmid.

The sgRNAs for the plasmid transfection were transcribed from a U6 promoter driven cassette, cloned into a pUC19, a pY108 (lenti-EnCpf1) or a pVax, as previously described [23–25].

### Cell lines

HEK293 cells were obtained from American Type Culture Collection (ATCC; www.atcc.org). HEK293-pCDNA-*NIPBL* c.5483G > A were produced by transfection of Bgl-II linearized pCDNA-*NIPBL* c.5483G > A plasmid in HEK293 cells. Cells were selected with 500 μg/ml of G418, 48 h after transfection. Single-cell clones (HEK293/CdLS-clones) were isolated and Sanger sequenced.

All HEK293 cells were cultured in Dulbecco's modified Eagle's medium (DMEM; Life Technologies) supplemented with 10% fetal bovine serum (FBS; Life Technologies), 10 U/ml antibiotics (PenStrep, Life Technologies) and 2-mM L-glutamine at 37 °C in a 5% CO2 humidified atmosphere. All cell lines were verified mycoplasma-free (PlasmoTest, Invivogen).

### Generation and culture condition of hiPSCs

Peripheral blood mononuclear cells (PBMCs) were donated by a patient diagnosed with CdLS with the informed consent of the parents. The research was approved by the internal ethical commitee of the Istituto di Ricerche Farmacologiche “Mario Negri”. PBMCs were freshly prepared from the patient blood sample, and were reprogrammed using CytoTune-iPS2.1 Sendai Reprogramming Kit (Invitrogen). Briefly, 5 × 10^5^ PBMCs were used for reprogramming and more than 20 iPSC-like cell colonies appeared after 3 weeks of culture. Out of these cell colonies, several symmetric and non-differentiated colonies were isolated and further expanded. Stemness of the isolated clones was confirmed in situ using Anti-TRA-1–60-Vio488 antibody (Miltenyi).

Patient-derived CdLS hiPSCs and commercial control hiPSC line (GIBCO, Thermo Fisher Scientific) were routinely cultured in Essential 8 (E8) medium or StemFlex medium (Thermo Fisher Scientific) and 10 U/ml antibiotics (PenStrep, Life Technologies) on Geltrex (Thermo Fisher Scientific)-coated plates (Costar) in a 5% CO_2_ humidified atmosphere medium. Medium was replaced every day or every other day. Cells were passaged every 3–4 days with EDTA-based dissociation solution.

### Transfection and electroporation of cell lines

HEK293 cells were transfected in 24-well multi-wells with 500–750 ng of Cas- or ABE-encoding plasmids, 250 ng of the desired pUC19-sgRNA plasmid and, in HDR experiments, 200–500 ng of ssODN using TransIT-LT1 (Mirus Bio), according to manufacturer’s instructions.

A total of 2 X 10^5^ HEK293/CdLS-clones (program CM-130, solution SE) and patient-derived hiPSCs (program CM-113, solution P3) were electroporated on a Lonza Nucleofector 4-D according to manufacturer’s instructions. Briefly, equal amount of 100-µM crRNA and tracrRNA (ordered from Integrated DNA Technologies) were mixed together to form gRNAs. 150 pmol of gRNAs were complexed with 120 pmol of Cas9 proteins (from Integrated DNA Technologies) to form RNPs. Electroporation mix was prepared as previously described [26]. When used, 1 µM of NU7441 (Selleck Chemicals, Cat# S2638) was added to the fresh medium on day 1 and day 2 after the electroporation.

### Detection of nuclease-induced genomic mutations

Genomic DNA was extracted using QuickExtract DNA extraction solution (Epicentre) and the target locus amplified by PCR using Phusion High Fidelity DNA Polymerase (Thermo Fisher). Oligos used to evaluate InDels resulting from cleavage of one gRNA are listed in Additional file 2: Table S2, purified PCR products were sequenced and analyzed using the TIDE, TIDER, EditR or the SYNTHEGO ICE software [27–30].

### In vitro differentiation by Embryoid Body (EB) assay

hiPSCs were collected and dissociated in order to obtain cell clumps, then plated in low attachment wells in 4 mL of fresh E8 medium supplemented with 10 μg/mL Y-27632 ROCK inhibitor (Tebu-BIO). Two days later, cell clumps were resuspended in 4 mL of 1:1 mix composed of Essential 6 medium (E6, Thermo Fisher Scientific) and E8 medium. Four days after, cell clumps were resuspended in 4 mL of 3:1 mix composed of fresh E6 medium and conditioned medium. Six days after, cell clumps were collected and resuspended in E6 medium (2.5:1.5 fresh/conditioned). At day 7, established EBs were collected and transferred on Geltrex-coated wells and cultured for further 7 days. In 0.5 mL of 1:1 mix composed of DMEM supplemented with 10% FBS and E6. Medium was changed every other day. For AFP and GATA4 marker analyses, EBs were cultured from day 14 to day 21 in DMEM supplemented with 10% FBS. Medium was changed every other day.

### RNA isolation, cDNA synthesis and quantitative polymerase chain reaction (qPCR)

RNA was purified with the NucleoSpin RNA kit (MACHEREY NAGEL) according to manufacturer’s instructions. cDNA was obtained by reverse-transcription with RevertAid First Strand cDNA synthesis kit (Thermo Fisher Scientific) and used to verify the expression of specific genes (primers reported in Additional file 2: Table S3). qRT-PCR was performed using HOT FIREPol EvaGreen qPCR Supermix (SOLIS BIODYNE), following the recommended protocol. Data were analyzed according to the comparative ΔΔCt method and normalized by using RPLP0 housekeeping gene.

### Immunofluorescence analysis

Cells were fixed in PFA 4% for 15 min at room temperature, permeabilized with 0.5% Triton X-100 for 15 min at room temperature and incubated in blocking solutions (0,3% Triton X-100, 5% FBS in PBS 1X) for 1 h at room temperature. Incubation with the primary antibodies was performed overnight at 4 °C in FBS 2%, Triton 0.2% with the antibodies reported in the Additional file 2: Table S3. The signal was revealed with the appropriate secondary antibodies (Additional file 2: Table S3). Nuclei were counterstained with Hoechst 33342 (1 μg/mL; Thermo Fisher Scientific). Pictures were detected with the microscope ZEISS Axio Observer and acquired with the camera Leica DFC450 C (Leica Microsystem).

### In silico off-target analysis

Off-targets for gRNA + 4 were analyzed by Cas-OFFinder online algorithm, by selecting: SpCas9 from *Streptococcus pyogenes*: 5’-NGG-3’, mismatch number ≤ 4, DNA bulge size = 0, RNA bulge size = 0 and as a target genome the *Homo sapiens* (GRCh38/hg38)—Human.

### GUIDE-seq and targeted Sanger sequencing

GUIDE-seq experiments were performed as previously described [31, 32]. Briefly, 2 × 10^5^ HEK293 cells were transfected using Lipofectamine 3000 transfection reagent (Invitrogen) with 500 ng of pxSpCas9, 250 ng of pUC19-sgRNA control or gRNA + 4 and 10 pmol of dsODNs. The day after transfection cells were detached and selected with 1 µg/ ml puromycin. Four days after transfection cells were collected and genomic DNA extracted using DNeasy Blood and Tissue kit (Qiagen) following manufacturer’s instructions and sheared using a Covaris S200 sonicator to an average length of 500 bp [31]. End-repair reaction was performed using NEBNext Ultra End Repair/dA Tailing Module and adaptor ligation using NEBNext® Ultra™ Ligation Module, as described by Nobles et al. [32]. Amplification steps were then performed following the GUIDEseq protocol from Tsai et al. [36].

Libraries were quantified with the Qubit dsDNA High Sensitivity Assay kit (Invitrogen) and sequenced with the MiSeq sequencing system (Illumina) using an Illumina Miseq Reagent kit V2-300 cycles (2 × 150 bp paired-end). Raw sequencing data (FASTQ files) were analyzed using the GUIDE-seq computational pipeline. GUIDE-seq data are listed in Additional file 3: Data—GUIDEseq.

#### Shallow whole genome sequencing (sWGS)

Genomic DNA of iPSCs was extracted using DNeasy Blood and Tissue kits (Qiagen) and DNA library was prepared using TruSeq PCR-Free Kit (Illumina, San Diego, CA) according to the manufactural protocol. DNA-sequencing was performed on the Illumina NextSeq500 with paired-end, 151 base pair long, reads. The overall quality of sequencing reads was determined using the FastQC protocol (https://www.bioinformatics.babraham.ac.uk/projects/fastqc/). Sequence alignments to the reference human genome (GRCh38) were performed using Burrows-Wheeler Alignment tool (BWA), a read alignment package that is based on a backward search with Burrows–Wheeler Transform (BWT), to efficiently align short sequencing reads against an extensive reference sequence such as the human genome, allowing mismatches and gaps [33]. The analysis was conducted in R using the software package QDNASeq [34], which implements a novel profile correction and blacklisting approach, in order to perform a downstream segmentation and calling of aberrations. The output of QDNASeq are the read counts per bin, which have been corrected, filtered, normalized, and optionally log2-transformed. The raw data are available in the Annotare database EMBL-EBI (https://www.ebi.ac.uk/fg/annotare/) under provisional accession number E-MTAB-11604 [35].

#### Flow cytometry analysis

Cells were dissociated using TrypLE (Gibco) and cell clumps were removed using a FACS strainer 40 mm (corning). Suspensions of single cells were directly incubated with conjugated antibody (Additional file 2: Table S4) diluted in PBS supplemented with 2% FBS for 20 min at 4 degrees. Samples were analyzed using the FACS Canto (at least 10,000 cells per sample) and cell sorting was performed using the FACS ARIA III (BD biosciences). Negative gates were set using isotype controls (Additional file 2: Table S3). Data were analyzed using FlowJo analysis software (BD biosciences).

#### Statistical analysis

All statistical analyses were performed using Prism 6 software to detect significant differences in measured variables among groups. A value of *P* < 0.05 indicates a statistically significant difference.

## Results

### Correction of the *NIPBL* c.5483G > A mutation in a HEK293-CdLS cell model

**Fig. 1:**
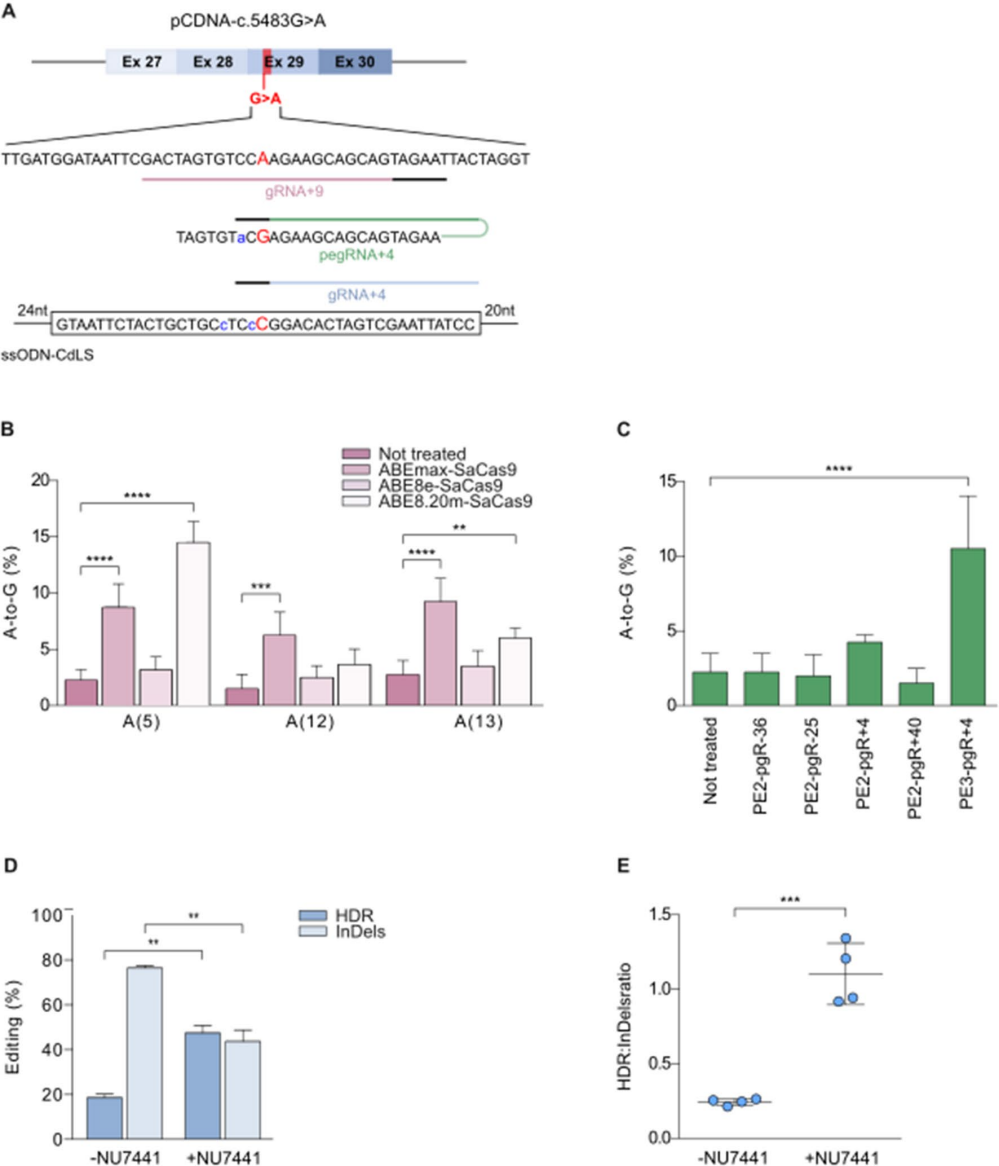
Correction of the NIPBL c.5483G > A substitution in a HEK293-CdLS cell model. **A** Scheme of the NIPBL cDNA (pCDNA–c.5483G > A) used to test the genome editing strategies for the G > A substitution (highlighted in red) in exon 29. The lower panel shows part of the nucleotide sequence and the target gRNA + 4, gRNA + 9 and pegRNA + 4 positions (underlined in pink, green and light blue, respectively, with the PAMs in black) and the ssODN-CdLS sequence (the corrected nucleotide is shown in red, silent mutations are shown in blue). **B** A-to-G transitions tested in HEK/CdLS clones (cl1 and 2) treated with ABEmax-SaCas9, ABE8e-SaCas9 and ABE8.20 m-SaCas9 combined with gRNA + 9. Modification of the adenines in the targeting window are reported numbered relative to the 5’ distal end of the gRNA, as reported by Rees et al. [17], A(5), A(12) and A(13). *n* ≥ 4 replicates. Data are means ± SD. Statistical analysis was performed using two-way ANOVA; **P ≤ 0.01, ***P ≤ 0.001, ****P ≤ 0.0001. **C** A-to-G transitions mediated by PE2 and PE3 strategies using the indicated pegRNAs (pgR + -25, pgRNA-36; pgRNA + 4; pgRA + 40) in HEK293/CdLS clones (cl1 and 2) through plasmid delivery; *n ≥ *2 replicates. Data are means ± SD. Statistical analysis was performed using two-way ANOVA; ****P ≤ 0.0001. **D** Editing efficiencies analyzed by TIDER in HEK293WT–CdLS cells (HEK293/CdLS-cl1) electroporated with SpHiFiCas9-gRNA + 4 RNPs and ssODN-CdLS untreated or treated with DN-PK inhibitor NU7441. **E** HDR/InDels ratio analyzed in cells treated as in **E**. Data were obtained from *n* = 4 experiments. Data are means ± SD. Statistical analysis was performed using two-way ANOVA; **P ≤ 0.01, ***P ≤ 0.001

To set up the correction strategy for the c.5483G > A mutation we generated HEK293 cell clones carrying the *NIPBL* coding sequence either wild-type or mutated. The *NIBPL* cDNA comprising exons 27–30 carrying the c.5483G > A mutation in exon 29, was stably integrated into HEK293 cells and two clones were isolated (HEK293/CdLS-cl1 and HEK293/CdLS-cl2). Sanger sequencing confirmed the presence of the c.5483G > A mutation in both clones (Fig. 1A, Additional file 1: Fig. S1A).

We initially tested the correction of the c.5483G > A mutation by using CRISPR base-editors, which have been developed to modify genomes in the absence of DSB [17, 36, 37]. We analyzed the protospacer adjacent motive (PAM) sequences 30 bp downstream from the mutated A, to select the best base-editor candidates to induce specific A to G transition. The PAM search was performed by taking into consideration the deaminase editing window which has been reported ranging between specific nucleotide positions with respect to the PAM^17^. We found no optimal PAM sequences (-NGG) for S*treptococcus Pyogenes* Cas9 (SpCas9), while we found a PAM (-NNGRRT) for *Staphylococcus Aureus* Cas9 with a compatible deaminase activity window to specifically modify the mutated A in *NIPBL* [17]. We thus designed a sgRNA (gRNA + 9) targeting a nickase SaCas9 adenine base-editor. We evaluated diverse versions of adenine deaminases (ABEmax, ABE8 and ABE8.20 m [38–40]) combined with nickase SaCas9 and gRNA + 9, by measuring the A to G transition. The mutated A nucleotide in position 12 with respect to the PAM (A12), which was located in the optimal predicted position for A to G transition, was minimally modified (almost 5% with the most efficient ABEmax), while we detected higher modifications (up to 14.5%) of a non-target A in position 5 (A5) from the PAM (Fig. 1B).

Since the base-editing approach did not produce substantial A to G reversion and bystander modification was significant on a non-target nucleotide (A5), we then tested the most recent CRISPR technology, prime-editing (PE), which similarly to base-editing allows to modify the genome without DSBs. To this aim, we designed four prime editing guide RNAs (pegRNAs, Additional file 2: Table S1) to apply both the PE2 and PE3 strategy as described by Anzalone et al.[18]. Even though the editing efficacy was higher than the one achieved with ABEs (up to 10.5% using the PE3 approach, Fig. 1C), the overall efficacy of c.5483G > A correction was not compatible with an application in primary cells which requires superior editing efficacy.

We then turned to the gene substitution approach using HDR induced by CRISPR-Cas9 nuclease activity in combination with a donor DNA sequence [41]. To identify the most efficient strategy we tested a variety of Cas9 orthologs having compatible PAMs which should be sufficiently close to the targeted mutation, ideally less than 10 bp distant [42, 43]. We found PAMs usable with SpCas9-NG, SpCas9-VQR, enAsCas12a and SaCas9 (Additional file 1: Fig. S1B) and compared their editing efficiency through formation of small insertions and deletions (InDels) in the HEK293/CdLS clones. The SpCas9-NG and Sp-Cas9 in combination with gRNA + 1 and gRNA + 4 targeting sequences near the c.5483G > A mutation, respectively, showed the highest editing rates with up to 42.5% InDels (Additional file 1: Fig. S1B-C and Additional file 2: Table S1). As donor we used a single strand oligonucleotide (ssODN-CdLS) carrying the correct *NIPBL* sequence and two additional silent mutations located in the seed region of the complementary sgRNA to prevent recutting after correction[44] (Fig, 1A and Additional file 2: Table S1). We initially attempted gene substitution through HDR by transfecting both the donor ssODN-CdLS and plasmids expressing the Cas9 nucleases. The HDR analysis revealed a higher correction efficiency by using SpCas9 in comparison with SpCas9-NG (7.2% and 2.5%, respectively), while the amounts of InDels generated by non-homologous end-joining (NHEJ) were 49.2% and 47.8%, respectively (Additional file 1: Figure S1D).

Since HDR efficiency is improved by ribonucleoprotein (RNP) delivery of CRISPR-Cas [45], we electroporated a high-fidelity version of SpCas9 recombinant protein, SpHiFiCas9 [46], along with a chemically synthesized gRNA. Strikingly, compared with plasmid transfection we obtained 2.6-fold improvement in HDR (18.7%) (Fig. 1D and E); as expected the InDels produced by NHEJ are higher (around four folds) than specific sequence substitution (Fig. 1D and E). To further enhance HDR, we tested NU7441, a compound that by blocking the NHEJ pathway through inhibition of the DNA-PK, favors HDR repair [26]. The HDR was further enhanced by the NU7441 resulting in 43.8% sequence substitution, thus at least twofolds more than untreated cells, while InDels generated by NHEJ decreased at similar levels as HDR (Fig. 1D and E).

Overall, these results suggest that the HDR strategy with SpHiFiCas9 and gRNA + 4 delivered as RNP together with ssODN-CdLS and the NU7441 treatment, is the most efficient method to correct the c.5483G > A mutation in the *NIPBL* gene in a HEK293-CdLS cellular models.

### Correction of the *NIPBL* c.5483G > A mutation in patient-derived hiPSCs

**Fig. 2:**
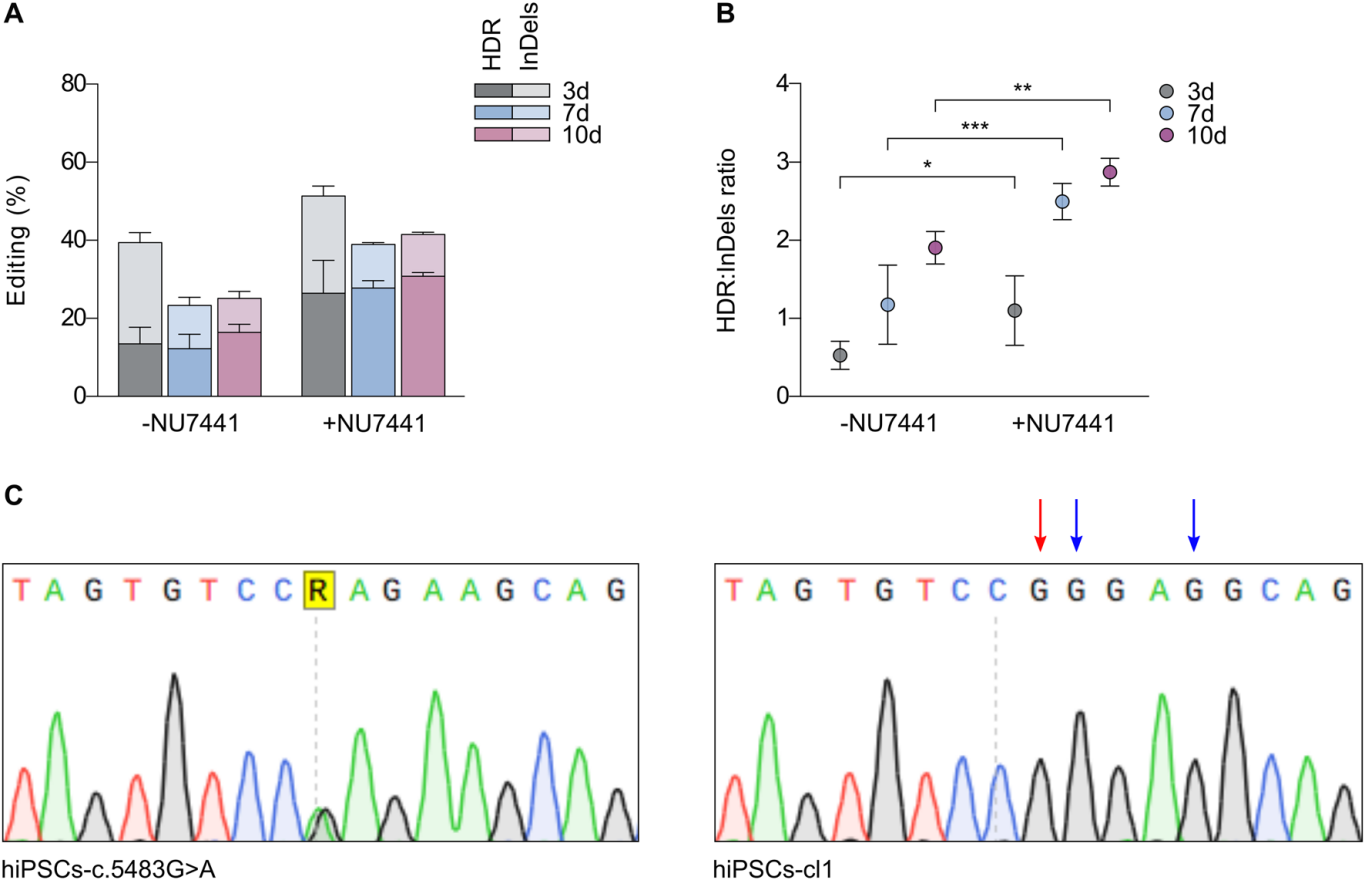
Correction of the NIPBL c.5483G > A substitution in hiPSCs derived from a CdLS patient. **A** Editing efficiencies analyzed by TIDER in hiPSCs-c.5483G > A electroporated with SpHiFiCas9-gRNA + 4 RNPs and ssODN-CdLS untreated (−) or treated ( +) with NU7441. **B** HDR/InDels ratio analyzed in cells treated as in **A**. **C** Sanger sequences of hiPSCs-c.5483G > A on the left and a fully corrected clone (hiPSCs-cl1) on the right. The red arrow indicates the target edit and blue arrows indicate the substitutions introduced in the ssODN to avoid Cas9 re-cleavage. Data were obtained from *n* ≥ 3 experiments. Data are means ± SD. Statistical analysis was performed using two-way ANOVA; *P ≤ 0.05, **P ≤ 0.01, ***P ≤ 0.001

Patient-derived hiPSCs are extensively used for disease modeling, drug screenings and somatic cell therapy [16, 47, 48]. Therefore, we generated hiPSCs from a CdLS patient carrying a c.5483G > A mutation in the *NIPBL* gene and then corrected the locus to generate isogenic wild-type and mutated cells. Gene correction was performed by electroporation of the mutated hiPSCs (hiPSCs-c.5483G > A) with SpHiFiCas9 and gRNA + 4 RNPs together with ssODN-CdLS and treated or not with NU7441. Editing efficiency in the bulk population was assessed after 3, 7 and 10 days, obtaining up to 16.4% of editing efficiency without NU7441, and up to 30.8% of editing efficiency with NU7441 after 10 days (Fig. 2A). Interestingly, HDR/InDels ratio increased with time, from 0.53 at day 3 to 1.9 and day 10 without NU7448, and from 1.1 at day 3 to 2.87 at day 10 with NU7448 (Fig. 2B).

To generate monoclonal edited derivatives, fourteen days after electroporation cells were sorted by flow cytometry using forward scatter and side scatter as parameters for the sorting. Single clones were expanded and Sanger sequencing analysis confirmed the presence of three fully corrected clones (hiPSCs-cl1/cl2/cl3) (Fig. 2C and Additional file 1: Fig. S2A).

### Characterization of edited hiPSC clones

**Fig. 3:**
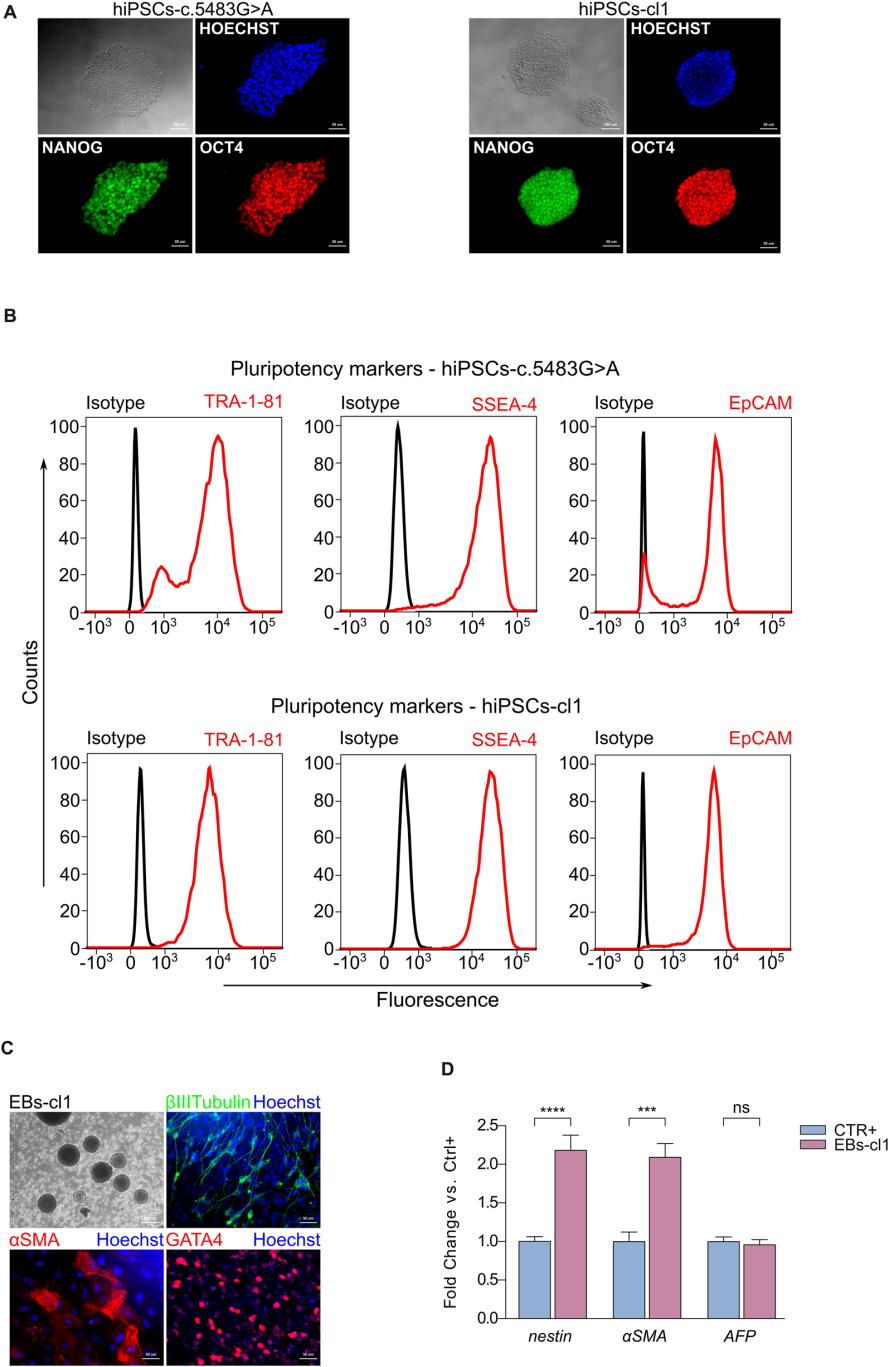
Characterization of pluripotent identity and competence of a corrected hiPSC clone. **A** Immunofluorescent staining for NANOG (green) and OCT4 (red) in hiPSCs-c.5483G > A (left) and hiPSCs-cl1 (right). Nuclei were stained with HOECHST (blue). **B** Flow cytometric analysis of membrane marker TRA-1–81, SSEA-4 and EpCAM in hiPSCs-c.5483G > A (top) and hiPSCs-cl1 (bottom). **C** Immunofluorescence staining showing the expression of marker genes belonging to the three germ layers in EBs obtained from hiPSCs-cl1. βIII-Tubulin (green), *α*SMA (red) and GATA4 (red). Nuclei were stained with HOECHST (blue). **D** qPCR analysis of the three germ layers markers nestin (ectoderm), *α*SMA (mesoderm) and AFP (endoderm) in EBs obtained from hiPSCs-cl1. EBs derived from commercial hiPSCs were used as positive control (CTR +). Data are means ± SD. Statistical analysis was performed using ordinary one-way ANOVA; ns *P* > 0.05, ***P ≤ 0.001, ****P ≤ 0.0001

Fully corrected and unmodified hiPSCs were expanded and analyzed for expression of pluripotency-associated markers. Immunofluorescence analysis confirms the expression of endogenous pluripotency markers, including NANOG and OCT4 in all three corrected clones and in control non-edited cells (Fig. 3A and Additional file 1: Fig. S2B). Moreover, we detected by flow cytometry high levels of surface pluripotency stem cell markers, including EpCAM, TRA-1-81 and SSEA-4 further confirming that the pluripotent status of the cells has been preserved during the editing treatments and clonal selection procedure (Fig. 3B and Additional file 1: Fig. S2C).

To functionally evaluate the pluripotency competence of the unmodified hiPSCs-c.5483G > A and the corrected hiPSC clones, we performed an Embryoid Body (EB) assay and checked for the expression of germ layers markers 14–21 days following their formation. Specifically, we found the presence of cells positive for βIII-Tubulin (ectodermal marker), αSMA (mesodermal marker) and GATA4 (endodermal marker), thus indicating the pluripotency of the hiPSCs we have generated (Fig. 3C, Additional file 1: Fig. S3A, C and E). The pluripotency competence was further quantitatively confirmed by assessing the level of expression of NESTIN (ectoderm), αSMA (mesoderm) and AFP (endoderm) transcripts (Fig. 3D, Additional file 1: Fig. S3B, D and F). As positive control for this analysis, EBs derived from commercially available hiPSCs were used (CTR+, see Material and Methods).

### Editing precision and genomic integrity of hiPSCs corrected via CRISPR-Cas9 technology

**Fig. 4:**
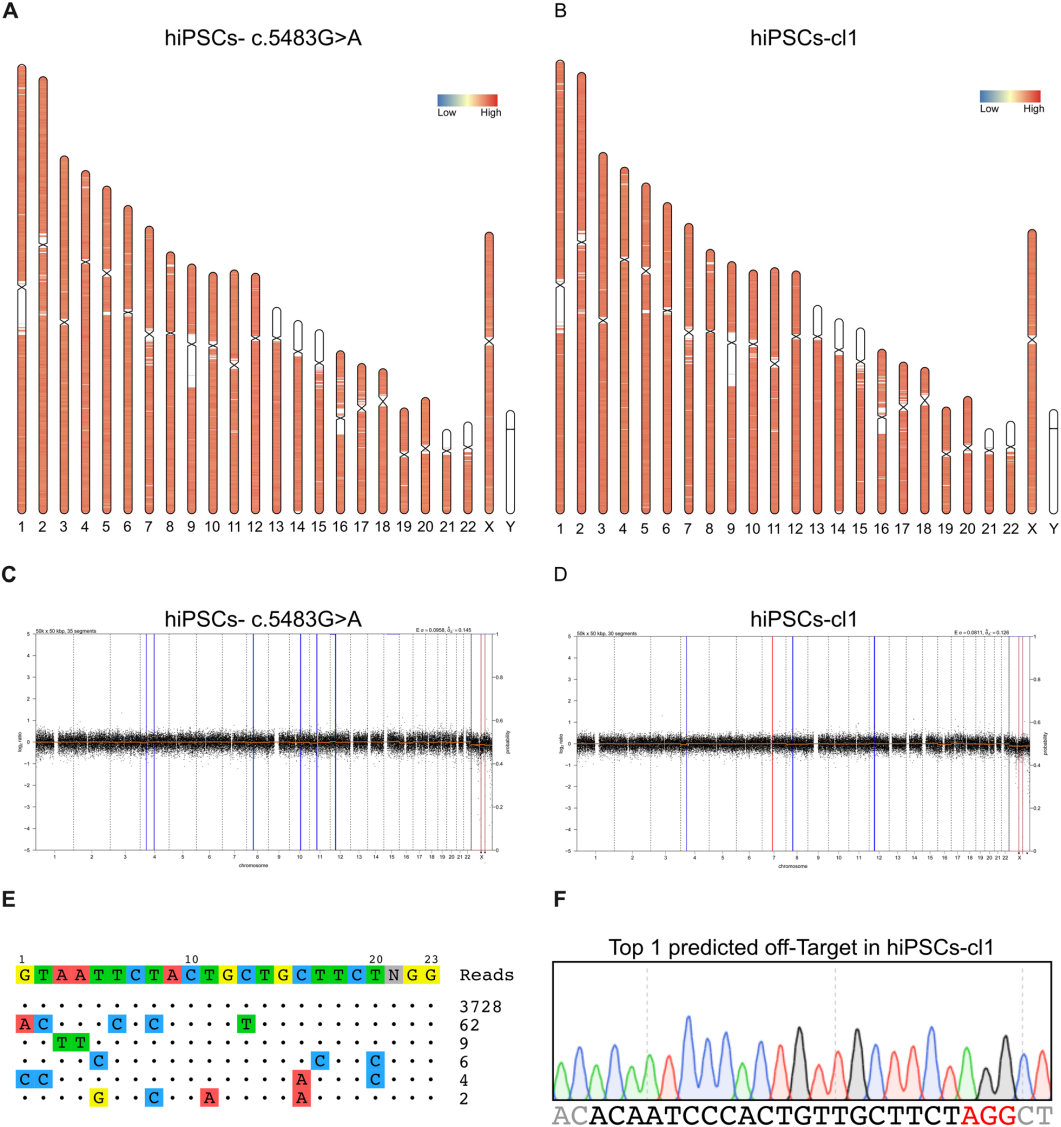
Karyotype, copy number variation (CNV) and precision of the CRISPR-Cas9 mediated NIPBL c.5483G > A correction in hiPSC-cl1. **A** Karyotype analysis conducted by carrying out a shallow Whole Genome Sequencing (sWGS) in hiPSCs-c.5483G > A. **B** Karyotype analysis conducted by carrying out a sWGS in hiPSCs-cl1. **C** CNV profile obtained by sWGS in hiPSCs-c.5483G > A. **D** CNV profile obtained by sWGS in hiPSCs-cl1. **E** GUIDE-seq analysis of gRNA + 4. **F** Sanger sequence of the top one predicted off-target of gRNA + 4 in hiPSCs-cl1. The protospacer is highlighted in black, PAM in red

To verify whether major genomic alterations may have occurred during editing of *NIPBL* locus and the expansion of the clones, DNA-seq of the entire genome was performed through shallow Whole Genome Sequencing (sWGS) in both hiPSCs-c.5483G > A and edited hiPSC clones. No substantial abnormalities could be observed in the karyotype, as well as in the copy number profile, between the hiPSCs-c.5483G > A and the edited clones (Fig. 4A–D and Additional file 1: Fig. S4A–D).

The potential off-target generated by the SpHiFiCas9-gRNA + 4 cleavages was assessed through the in silico analysis (Cas-OFFinder [49]) and a genome wide assay, the GUIDE-seq method[31]. The off-target prediction performed through the Cas-OFFinder software revealed 188 potential off-targets from 1 to 4 mismatches (Additional file 2: Table S2). The experimental off-target genome-wide analysis was performed through GUIDE-seq in HEK293 treated with SpCas9-gRNA + 4 and showed mainly on-target cleavages and near background levels of unpredicted cuts (5 sites) (Fig. 4E). The five unpredicted cleaved sites showed near back-ground cleavages (sequence reads below 10) apart for one that even though appeared with much less sequence reads than the on target, was higher than the other sites (62 sequence reads) (Fig. 4E). To verify the potential modification of the most represented off-target site, we performed Sanger sequencing in the corrected hiPSC clones. The sequencing results showed that the potential off-target is not altered in the modified hiPSCs thus indicating a precise editing protocol (Fig. 4E-F and Additional file 1: Fig. S4E–F).

## Discussion

CdLS is a severe genetic disorder characterized by a large spectrum of phenotypes, including systemic malformations, organ system manifestations, cognitive and behavioral dysfunctions [2, 50]. There is increasing evidence that mutations causing CdLS generate alterations in important biological processes, including gene regulations, DNA repair and translation [51]. Understanding the molecular mechanisms at the base of CdLS is fundamental to design specific therapeutic strategies. For example, deregulation of the canonical WNT pathway has been proposed to be linked to CdLS malformations mainly due to developmental impairment [15, 52]. These studies demonstrate that lithium chloride (LiCl) activates this pathway thus rescuing morphological neural defects in *nipblb* knockdown zebrafish and in a *Drosophila melanogaster* CdLS model, by restoring the physiological level of proliferation and neural differentiation of CdLS neural stem cells [53, 54]; similar proliferation effects were also observed in patient derived lymphoblastoid cell lines [53, 54]. hiPSCs represent a powerful cellular model to gain deep knowledge on disease molecular determinants and for the identification of therapeutic targets for the development of pharmaceutical intervention. For example, RNA-sequencing of *NIPBL* haploinsufficiency in hiPSCs and in vitro derived cardiomyocytes allowed the identification of hundreds of transcripts with altered expression, including dysregulated genes responsible for the normal development of the heart [14].

CRISPR-Cas technologies are powerful tools to modify genomes for therapeutic purposes and to generate specific disease models [55]. We tested the most recent techniques using DSB free approaches including base- and prime-editing [18, 40] and compared them with Cas9 nuclease approach to induce gene substitution through HDR [26, 41]. Among the base-editor limitations are the PAM sequence selection which is limited to the window of the deamination activity and the off-target deamination which may induce modifications beyond the target nucleotides (bystander edits). Indeed, our base-editor choice was restricted to one type of ABE-SaCas9 constrained by the available PAM sequences surrounding the mutation which however determined bystander modifications and inefficiently repair the target mutation. Additional ABEs with shifted editing windows or using different Cas variants that have been recently generated may overcome these problems [56, 57]. Although very promising, the efficiency of the prime-editor technology still highly depends on the target locus and on the case of the *NIPBL* gene did not appear particularly suitable.

We found that the most efficient and precise strategy to correct the *NIPBL* c.5483G > A mutation in hiPSCs was the delivery of a high-fidelity version of SpCas9 protein complexed with a gRNA to promote HDR using a ssODN as donor template. Repair via HDR is reported to be inefficient and the outcome of the DSB promoted by Cas nucleases often results in high InDels, because the error prone NHEJ pathway is favored [58]. However, several methodologies have been described to enhance HDR efficiency and to increase HDR/InDels ratio, including the chemical compound NU7441 [59, 60]. Using this molecule, we reached up to 30.8% of editing efficiency and a 2.87 HDR/InDels ratio in patient derived hiPSCs. The efficient editing facilitated the isolation of hiPSC clones carrying the programmed modification and with preserved staminal properties. Interestingly, HDR/InDels ratio increased over time, likely due to depletion of hiPSCs harboring disrupted *NIPBL* in both alleles. This hypothesis is sustained by the lack of homozygous knock out clones that would be generated by InDels in both alleles, strongly suggesting the lethality of *NIPBL* insufficiency in hiPSCs.

We demonstrated that the isolated hiPSC clones with the desired A to G correction retained the pluripotent properties and showed no major genomic defect; additionally, no off-target cleavages were detected by sequencing analysis of the top potential off-target sites revealed by the whole genome approach GUIDE-seq.

Since CdLS pathogenesis include differentiation impairment at the embryonal stage [61] and involves various cell types, hiPSCs with their multi-lineages differentiation properties offer considerable advantages to study the molecular mechanism leading to CdLS and develop therapeutic strategies. Importantly, the isogenic hiPSC landscape generated through CRISPR-Cas technology provide a better controlled experimental setup.

## Conclusions

With this study, we identified the most efficient and precise genome editing strategy using CRISPR-Cas technology to repair a mutation in the *NIPBL* gene which causes CdLS. The strategy was validated in patient-derived hiPSCs which have been confirmed for genetic integrity and staminal properties. The derived wild-type and mutated isogenic hiPSC clones provide a valuable cellular model to advance knowledge on the molecular events leading to CdLS and advancement toward therapeutic strategies.

## Supplementary Information

**Additional file 1**. Supplementary Figures.

**Additional file 2**. Supplementary Tables.

**Additional file 3**. Data—GUIDEseq.

## Data Availability

Data and materials are reported in the manuscript in main text and supplementary information.

## Abbreviations

ABE: Adenine Base Editing/Adenine Base Editor
BWA: Burrows-Wheeler Alignment
CdLS: Cornelia de Lange Syndrome
CRISPR: Clustered Regularly Interspaced Short Palindromic Repeats
DBS: Double Strand Break
E8: Essential 8
EB: Embryoid Body
HDR: Homology Directed Repair
hiPSC: human-induced Pluripotent Stem Cell
InDels: Insertions and Deletions
NHEJ: Non-Homologous End Joining
NIPBL: Nipped-B-like protein
PAM: Protospacer Adjacent Motive
PBMC: Peripheral Blood Mononuclear Cells
PE: Prime Editing
RNP: Ribonucleoprotein
ssODN: single strand Oligonucleotide
sWGS: shallow Whole Genome Sequencing
