# Supplementary material for: Generation of corrected hiPSC clones from a Cornelia de Lange Syndrome (CdLS) patient through CRISPR-Cas-based technology
Source: Stem Cell Res Ther. 2022 Sep 2;13:440. doi: 10.1186/s13287-022-03135-0 (PMC9438151; doi:10.1186/s13287-022-03135-0)
Supplement: Supplementary file 1 — Additional file 1. Supplementary Figures. [file 13287_2022_3135_MOESM1_ESM.docx]

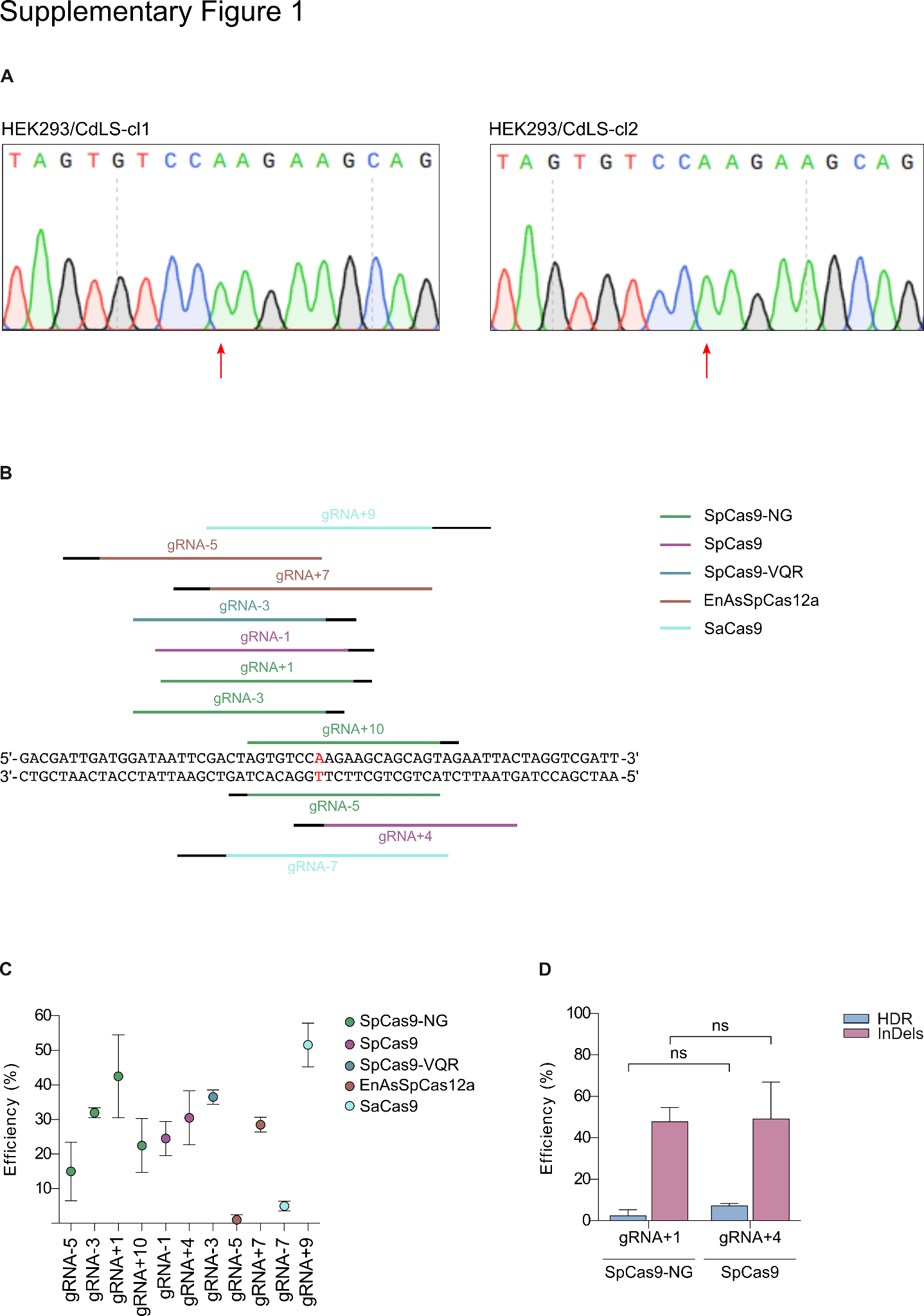


***Supplementary Figure 1. ABE and PE strategies to correct the NIPBL c.5483G>A substitution.***

***A)*** *Sanger sequences of HEK/CdLS-cl1 and HEK/CdLS-cl2.* ***B)*** *Schematic of gRNAs designed at NIPBL locus near the c.5483G>A mutation. Colors indicate targeted Cas variants, PAMs are highlighted in black.* ***C)*** *Activity of gRNAs at NIPBL locus near the c.5483G>A substitution; n****≥****2 replicates. Data are means ±SD.* ***D)*** *Editing efficiencies in HEK293/CdLS clones transfected with plasmids encoding SpCas9-NG or SpCas9, gRNA+1 or gRNA+4 and ssODN-CdLS; n=6 replicates. Data are means ±SD. Statistical analysis was performed using two-way ANOVA; nsP>0.05.*


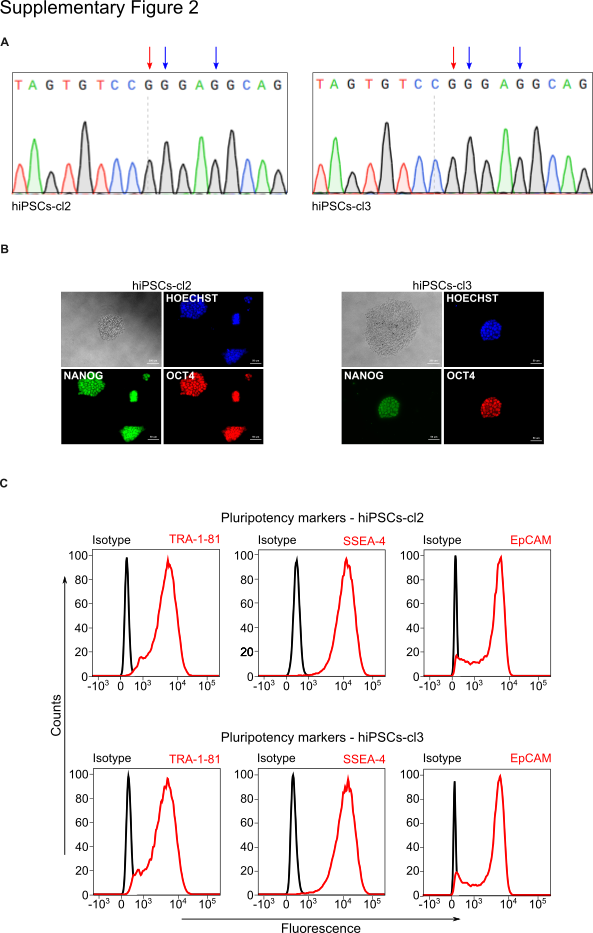


***Supplementary Figure 2. Characterization of pluripotent identity of hiPSCs-cl2 and hiPSCs-cl3.
A)*** *Sanger sequences of hiPSCs-cl2 and hiPSCs-cl3 at the NIPBL c.5483 locus.* ***B)*** *Immunofluorescent staining showing the expression of the stemness marker protein NANOG (green) and OCT4 (red) in hiPSCs-cl2 (left) and hiPSCs-cl3 (right). Nuclei have been stained with HOECHST (blue).* ***C)*** *Flow cytometric analysis showing the expression of superficial stem cell marker protein TRA-1-81, SSEA-4 and EpCAM in hiPSCs-cl2 (top) and hiPSCs-cl3 (bottom).*


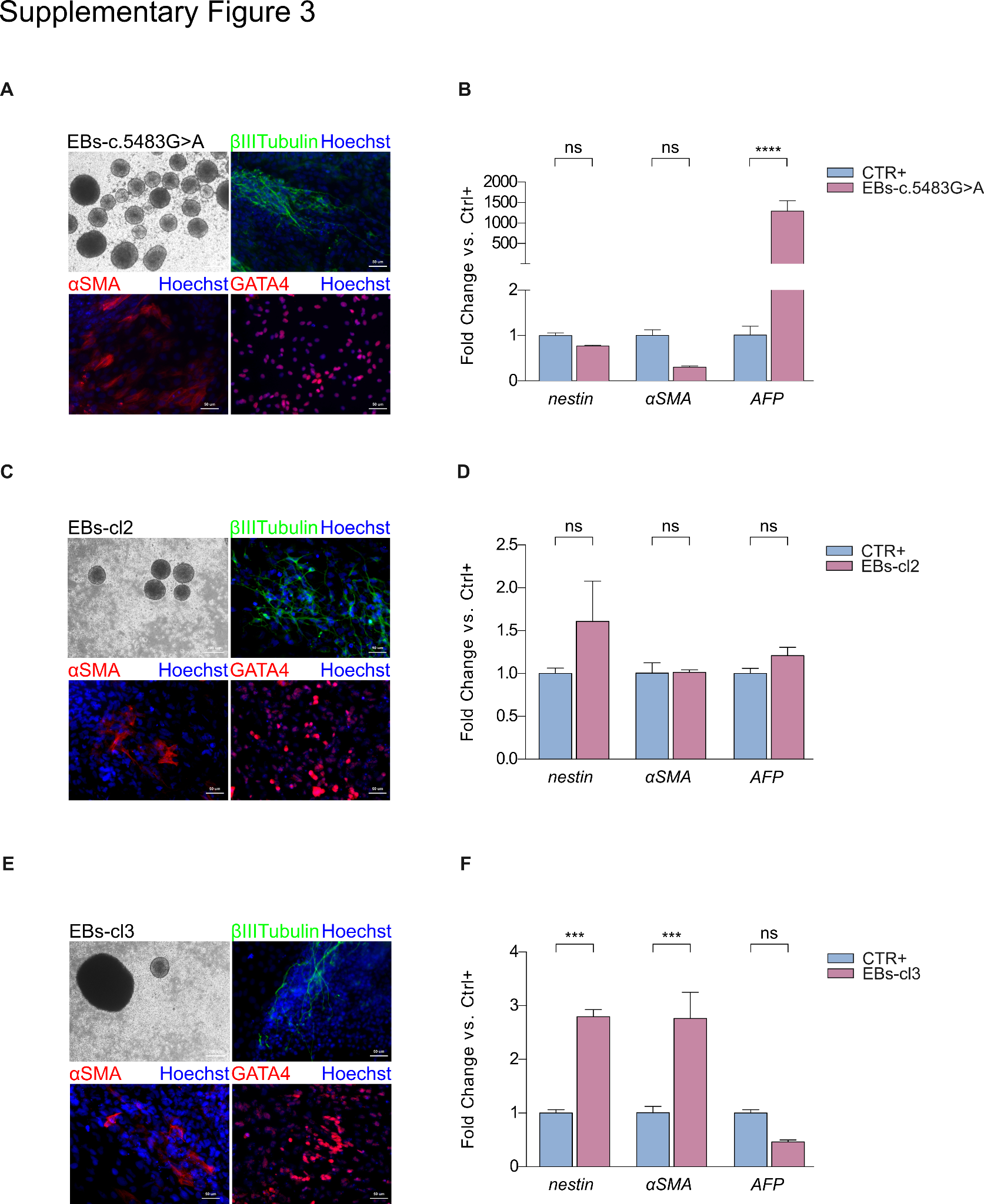


***Supplementary Figure 3. Three germ layer cell derivatives in EBs derived from hiPSCs-cl2 and hiPSCs-cl3.***

***A, C*** *and* ***E)*** *Immunofluorescence staining showing the protein expression of marker genes belonging to the three germ layers in embryoid bodies obtained from unmodified hiPSCs-c-5483G>A, hiPSCs-cl2 and hiPSCs-cl3. βIII-Tubulin (green), αSMA (red) and GATA4 (red). Nuclei have been stained with HOECHST (blue).* ***B, D*** *and* ***F)*** *qPCR analysis evaluating the expression of the three germ layers marker nestin (ectoderm), αSMA (mesoderm) and AFP (endoderm) in embryoid bodies obtained from unmodified hiPSCs-c.5483G>A, hiPSCs-cl2 and hiPSCs-cl3. Commercial hiPSCs were used as positive control (CTR+). Data are means ±SD. Statistical analysis was performed using ordinary on-way ANOVA; nsP>0.05, ***P*≤*0.001, ****P≤0.0001.*


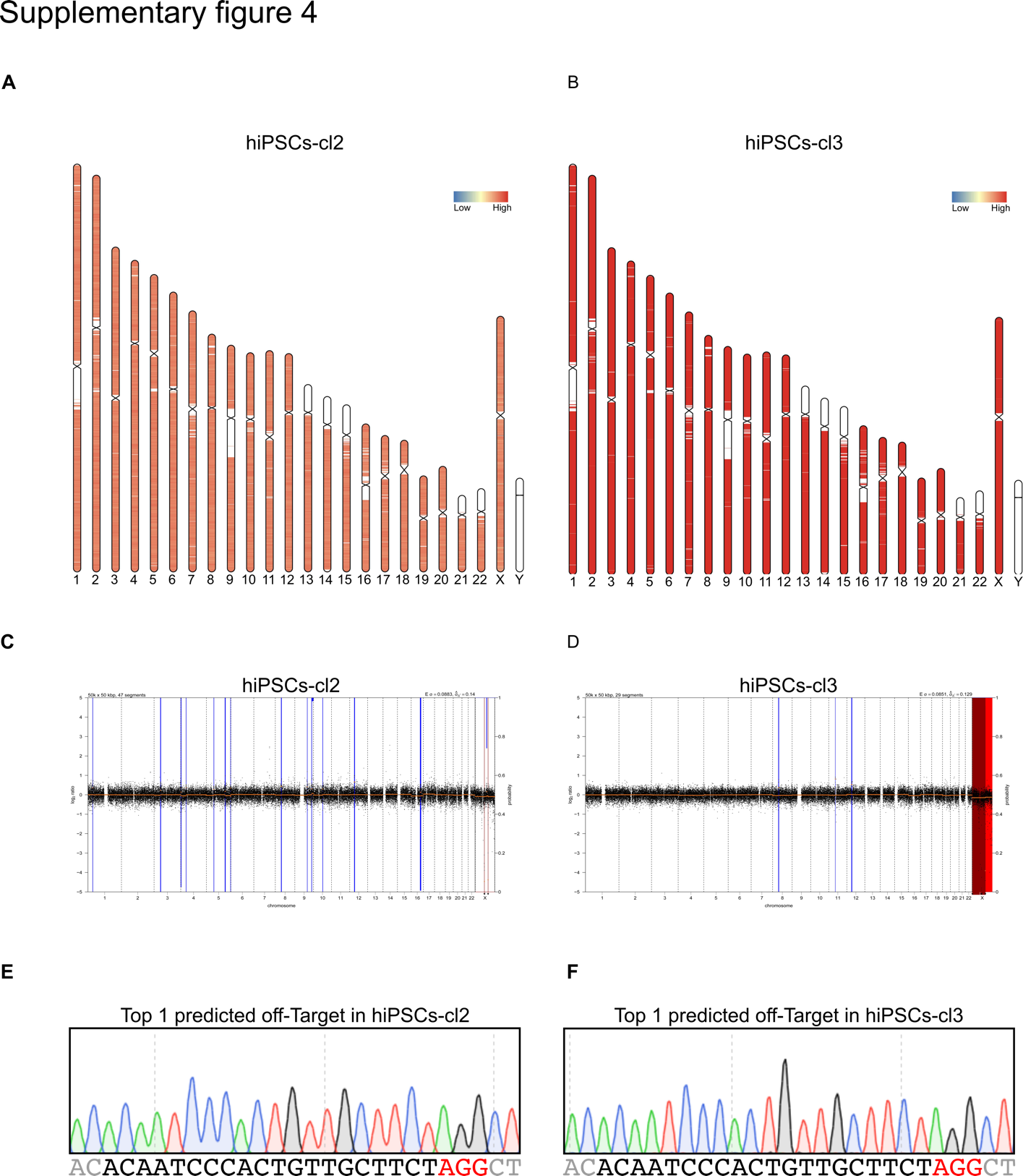


***Supplementary Figure 4. Karyotype, copy number variation (CNV) and precision of the CRISPR-Cas9 mediated NIPBL c.5483G>A correction in hiPSCs-cl2 and hiPSCs-cl3.***

***A)*** *Karyotype analysis conducted by carrying out a shallow Whole Genome Sequencing (sWGS) in hiPSCs-cl2.* ***B)*** *Karyotype analysis conducted by carrying out a sWGS in hiPSCs-cl3.* ***C)*** *CNV profile obtained by sWGS in hiPSCs-cl2.* ***D)*** *CNV profile obtained by sWGS in hiPSCs-cl3.* ***E-F)*** *Sanger sequences of the top 1 predicted off-Target of gRNA+4 in hiPSCs-cl2 and hiPSCs-cl3. The protospacer is highlighted in black, PAM in red.*
